# Is Neutrophil/Lymphocyte Ratio Associated with Subclinical Inflammation and Amyloidosis in Patients with Familial Mediterranean Fever?

**DOI:** 10.1155/2013/185317

**Published:** 2013-06-20

**Authors:** Ali Ugur Uslu, Koksal Deveci, Serdal Korkmaz, Bahattin Aydin, Soner Senel, Enver Sancakdar, Mehmet Sencan

**Affiliations:** ^1^Department of Internal Medicine, Faculty of Medicine, Cumhuriyet University, 58140 Sivas, Turkey; ^2^Department of Medical Biochemistry, Faculty of Medicine, Cumhuriyet University, 58140 Sivas, Turkey; ^3^Department of Hematology, Faculty of Medicine, Cumhuriyet University, 58140 Sivas, Turkey; ^4^Department of Rheumatology, Faculty of Medicine, Erciyes University, Kayseri, Turkey

## Abstract

*Background*. The purpose of the present study is to determine the association between neutrophil/lymphocyte ratio and both subclinical inflammation and amyloidosis in familial Mediterranean fever. *Methods*. Ninety-four patients with familial Mediterranean fever and 60 healthy volunteers were included in the study. Of the patients, 12 had familial Mediterranean fever related amyloidosis. The neutrophil/lymphocyte ratio of the patients was obtained from the hematology laboratory archive. *Results*. The neutrophil/lymphocyte ratio was significantly higher among persons with familial Mediterranean fever compared to healthy individuals (*P* < 0.0001). Also, neutrophil/lymphocyte ratio was significantly higher in patients with amyloidosis than in amyloidosis-free patients (*P* < 0.0001). Since NLR was evaluated in nonamyloid and amyloid stages of the same patient population (type 1 phenotype), we obtained significant statistical differences (1.95 ± 0.30 versus 2.64 ± 0.48, *P* < 0.05, resp.). With the cutoff value of neutrophil/lymphocyte ratio >2.21 and AUC = 0.734 (*P* = 0.009), it was a reliable marker in predicting the development of amyloidosis. *Conclusion*. The neutrophil/lymphocyte ratio, an emerging marker of inflammation, is higher in patients with familial Mediterranean fever in attack-free periods. The neutrophil/lymphocyte ratio may be a useful marker in predicting the development of amyloidosis.

## 1. Introduction 

Familial Mediterranean fever (FMF) is a genetic disease with autosomal recessive inheritance and is characterized by acute episodes of serosal membrane inflammation and increased risk of renal amyloidosis [1]. The clinical disease is common in Turks, Armenians, Arabs, and non-Ashkenazi Jews [2]. The FMF gene, also called as Mediterranean Fever (MEFV) gene, was mapped to the short arm of chromosome 16. This gene encodes pyrin/marenostrin. The attack periods of FMF last in 1–3 days. The erythrocyte sedimentation rate (ESR) and acute phase proteins such as C-reactive protein (CRP), serum amyloid A (SAA), and fibrinogen increase during the attack periods and usually return to normal in attack-free periods [3, 4]. The attack periods and complications of FMF are managed with colchicine therapy.

It is known that subclinical inflammation continues during the attack-free period in patients with FMF [5]. This type of subclinical inflammation has a chronic course and leads to the development of the most mortal complication named amyloidosis. And amyloidosis can be controlled with the inhibition of this inflammation [6].

The neutrophil and lymphocyte counts can be obtained with a basic hemogram test. The neutrophil/lymphocyte ratio (NLR) may be an indicator of systemic inflammation [7]. NLR has been associated with some conditions such as chronic inflammation in cardiovascular diseases, malignancies, ulcerative colitis, and hepatic cirrhosis, and it has been suggested that NLR has a prognostic importance [8, 9].

We conducted the present study to determine the association between NLR and both subclinical inflammation and amyloidosis in familial Mediterranean fever.

## 2. Materials and Methods

Ninety-four patients with FMF, who met the Tel-Hashomer criteria at the Rheumatology Clinic of the School of Medicine of the Cumhuriyet University (Sivas, Turkey) between January 2010 and August 2011, were evaluated by a retrospective review of records. Sixty age- and sex-matched control participants were included in the study. The Ethics Committee for Clinical Research of Cumhuriyet University School of Medicine approved this study.

The patients with diabetes mellitus, coronary heart diseases, metabolic syndrome, anemia, acute/chronic infection, autoimmune disorders, chronic obstructive pulmonary disease, and history of smoking were excluded. Also, the patients under medication except colchicine were not included in the study. The clinical symptoms, laboratory values, and MEFV gene mutations were achieved from the archives records.

Of the 94 patients, all were in attack-free period, and those patients had been followed up regularly. At least 2 weeks from the end of an FMF attack period was described as attack-free period according to the physical examination, clinical symptoms, and acute phase proteins such as CRP, fibrinogen, and leucocyte counts. The white blood cell, neutrophil, and lymphocyte counts were recorded, and the NLR was calculated from these parameters. 

The diagnosis of amyloidosis was made by renal or rectal biopsy. Of the patients, 12 were diagnosed as amyloidosis. In amyloidosis group, 9 patients had type 1 phenotype, and 3 patients had type 2 phenotype.

### 2.1. Statistical Analyses

All statistical analyses were performed with the Statistical Package for the Social Sciences (SPSS) 15.0 Package (SPSS Inc., Chicago, IL, USA). Descriptive statistics were presented as arithmetic mean ± standard deviation. The significance of the mean differences between groups was assessed by Student's *t*-test and Mann-Whitney *U* test. Also, the nonparametric Wilcoxon Signed Ranks test was used to test for differences between related (paired) samples. Relationships between variables were tested using Pearson's correlation analysis. ROC curve graphics were used in the comparison of sensitivity and specificity. *P* values of less than 0.05 were regarded as significant. 

## 3. Results

There was not statistical differences between patients with FMF and healthy individuals in terms of age (29.9 ± 12.2 versus 31.3 ± 9.4 years, resp.). And also, we did not obtain statistical differences between patients with FMF and controls in terms of gender (M/F = 30/64 versus M/F = 20/40, resp.). The baseline clinical characteristics of FMF patients were summarized in Table 1. And also, baseline laboratory characteristics of patients and controls were displayed in Table 2.

 NLR was significantly higher in FMF patients than the control group (2.06 ± 0.61 versus 1.59 ± 0.42, resp.; *P* < 0.0001). NLR was significantly higher in patients with FMF related amyloidosis than the controls (2.51 ± 0.62 versus 1.59 ± 0.42, resp.; *P* < 0.0001). NLR was significantly higher in patients with amyloidosis than in patients with amyloidosis free (2.51 ± 0.62 versus 1.99 ± 0.58, resp.; *P* < 0.05). Since NLR was evaluated in nonamyloid and amyloid stages of the same patient population (type 1 phenotype = 9 patients), we obtained significant statistical differences (1.95 ± 0.30 versus 2.64 ± 0.48, *P* < 0.05, resp.). The detailed results are outlined in Table 3. 

No significant differences were found between NLR and the ages of patients, gender of patients, recurrent attacks, duration of attacks, duration of the illness, severity of the illness, acute phase proteins such as ESR and CRP and MEFV gene mutations (*P* > 0.05). 

**Figure 1:**
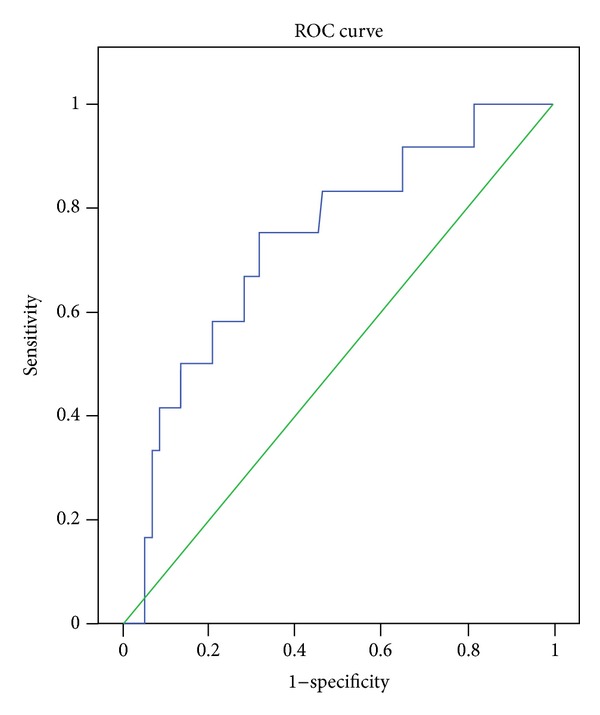
The ROC curve showing the performance of NLR in patients with FMF.

Since NLR showed an AUC of 0.734 (95% CI, 0.585–0.884) with a cutoff value of 2.21 by ROC analysis, it was a reliable marker in predicting the development of amyloidosis. This relationship was displayed in Figure 1.

## 4. Discussion

Familial Mediterranean fever (FMF) is an inherited autosomal recessive disorder, ethnically restricted and commonly found among individuals of Mediterranean descent, caused by MEFV gene mutations on chromosome 16. MEFV gene has over 218 mutations, and these mutations have been identified mostly in exon 2 (E148Q) and exon 10 (M694V, M694I, V726A, M680I) [11]. 

The MEFV gene encodes pyrin/marenostrin. Pyrin inhibits the proinflammatory cytokines and/or increases the secretion of anti-inflammatory mediators. The downregulation of pyrin leads to microtubule activation and/or the migration of inflammatory cells in inflamed tissues [12, 13]. In this process, loss of pyrin may result in increase in interleukin-1 beta (IL-1β), interleukin-6 (IL-6), and tumor necrosis factor-alpha (TNF-*α*) release, so an acute phase response is generated. As a consequence, acute phase proteins such as CRP and SAA are produced in large amounts by liver in response to inflammation [3, 4].

The subclinical inflammation continues in up to 30% of the patients during the attack-free periods of FMF. The testing of proinflammatory cytokines and SAA can demonstrate this subclinical inflammation [14, 15]. Yildirim et al. [16] demonstrated that IL-1β levels are increased in FMF patients during attack-free period, and serum IL-1β values seem to correlate with CRP levels. So, the elevation of IL-1β levels may be important in monitoring subclinical inflammation of attack-free period in FMF patients. And also, Kiraz et al. [17] demonstrated that IL-6, IL-8, and TNF-*α* levels were observed in FMF patients, which could reflect the presence of sustained inflammation in attack-free FMF patients. 

In a significant proportion of patients with FMF, SAA remains elevated during attack-free periods, thereby increasing the risk of developing amyloidosis. Berkun et al. [14] stated that elevated SAA levels are found in a third of FMF patients during an attack-free period. Measurement of SAA level may help in the diagnosis of FMF and in adjustment of the colchicine dose because SAA measurement led to a change in colchicine dose in 30% of the patients in their study.

NLR was measured by dividing neutrophil count to lymphocyte count. In an acute setting, lymphopenia is a common finding during a stress response secondary to increased levels of corticosteroids [18]. And also, lymphopenia is observed in inflammatory states due to increased lymphocytes apoptosis [19].

NLR has been shown as an indicator of systemic inflammation in various conditions [7]. For example, NLR has been associated with poor outcomes in patients with cardiovascular diseases. Demir [20] suggested that higher NLR has a positive correlation with blood pressure and is elevated in nondippers compared with dippers. It has been recently shown that inflammation may play a role in myocardial ischemia. Muhmmed Suliman et al. [21] suggests that NLR provides a simple and inexpensive method for assessment of inflammatory status in patients with acute coronary syndrome. 

Many cancer survival studies have suggested that NLR is a significant predictor of overall and disease specific survival of patients [22–24]. In malignancies, systemic inflammation was thought to be secondary to tumor hypoxia or necrosis and related with antiapoptosis [25]. NLR was an index of systemic inflammation [26], and prognostic value had been studied in many types of cancer, including nonsmall cell lung cancer [27], colorectal cancer [22], breast cancer [28], and gastric cancer [29].

Most of the recent studies showed that there was a correlation between inflammatory mediators (IL-6, TNF-*α*) and components of metabolic syndrome (MS) [30]. Buyukkaya et al. [31] concluded that there is a significant correlation between the criteria of MS and inflammation on the basis of NLR. And they suggest an increase in NLR as the severity of MS increases. 

Ulcerative colitis (UC) is a chronic inflammatory disease causing continuous mucosal inflammation. It is important to determine disease activity early as this will significantly reduce the surgery rate and therefore reduce mortality in patients with serious UC. Nevertheless, an optimal test has not yet been developed. Celikbilek et al. [32] showed that NLR is higher in patients with active UC compared with controls and UC patients in remission, and a cutoff value of 2.47 can be used to identify patients with active UC.

The typical clinical course of FMF is characterized by bouts of painful inflammation, but in many patients inflammation can persist in attack-free periods (in up to 30% of the patients), as shown by high levels of acute phase proteins and cytokines [3, 15]. This subclinical inflammation increases the risk of developing complications such as anemia, splenomegaly, decreased bone mineral density, heart disease, and life-threatening amyloidosis. Lifelong treatment with colchicine is required to prevent the inflammatory attacks and the deposition of amyloid because renal amyloidosis can be prevented by colchicine.

In our study, we found that NLR was significantly higher in patients with FMF compared to healthy individuals. Amyloidosis, which can lead to renal failure, is the most severe complication of FMF. As is known, amyloidosis is a consequence of longstanding inflammation. With the cutoff value of NLR > 2.21, it was a reliable marker in predicting the development of amyloidosis in our study because NLR was significantly higher in patients with FMF related amyloidosis than in patients with amyloidosis-free FMF. Since NLR was evaluated in nonamyloid and amyloid stages of the same patient population (type 1 phenotype = 9 patients), we have shown that NLR is higher in amyloid stage than the nonamyloid stage. 

In conclusion, NLR is an emerging marker of inflammation and is higher in patients with FMF in attack-free periods. NLR is an important measure of systemic inflammation as it is cost effective, readily available, and can be calculated easily. Our study demonstrates that NLR is strongly associated with attack-free periods in patients with FMF. We suggest that NLR may show subclinical inflammation in patients with FMF in attack-free periods, and NLR may be a useful marker in predicting the development of amyloidosis. That we could not compare NLR with other inflammatory cytokines and SAA simultaneously to show the accuracy of the NLR for detecting subclinical disease was the limitation of our study. Future studies are needed to externally cross-validate our findings in a larger cohort of FMF patients. 

## Figures and Tables

**Table 1:** Baseline characteristics of patients with FMF.

	Patients *n* = 94
Age at diagnosis, (years)*	23.1 ± 12.8
Duration of illness, (years)*	6.6 ± 7.3
Family history of FMF, (*n*, %)	54 (57.4)/42 (42.6)
Dose of colchicine, (mg/day)*	1.43 ± 0.55
Response to colchicine, (*n*, %)	88 (93.6)/6 (6.4)
Scoring system of Pras et al. [10] (mild, moderate, severe)	5.44 ± 2.27
Fever, (*n*, %)	87 (92.6)
Peritonitis, (*n*, %)	87 (92.6)
Pleuritis, (*n*, %)	58 (61.7)
Pericarditis, (*n*, %)	8 (8.5)
Arthritis, (*n*, %)	16 (17)
Arthralgia, (*n*, %)	55 (58.5)
Myalgia, (*n*, %)	44 (46.8)
Erysipelas-like erythema, (*n*, %)	6 (6.4)
MEFV mutations, (*n*, %)	
Homozygous M694V	9 (9.6)
Heterozygous M694V	26 (27.7)
Heterozygous M680I	11 (11.7)
Heterozygous E148Q	11 (11.7)
Heterozygous V726A	4 (4.3)
Heterozygous M694V/M680I	4 (4.3)
Heterozygous M694V/E148Q	3 (3.2)
Others	26 (27.7)

*Mean ± SD; [10] is reference number in the references section.

**Table 2:** Baseline laboratory characteristics of patients and controls.

	Patients *n* = 94 (mean ± SD)	Controls *n* = 60 (mean ± SD)	*P* value
Hemoglobin, g/dL	13.59 ± 1.96	14.37 ± 1.45	0.005
Leucocyte, ×10^9^/L	7.34 ± 1.90	6.81 ± 1.43	0.520
Platelet, ×10^9^/L	287.25 ± 84.53	271.36 ± 54.72	0.159
Neutrophil, ×10^9^/L	4.42 ± 1.22	3.72 ± 0.96	<0.0001
Lymphocyte, ×10^9^/L	2.25 ± 0.73	2.37 ± 0.65	0.280
NLR, %	2.06 ± 0.61	1.59 ± 0.42	<0.0001
ESR, mm/h	11.34 ± 10.32	6.40 ± 4.13	<0.0001
CRP, mg/L	3.17 ± 2.11	2.43 ± 1.57	0.380

NLR: neutrophil/lymphocyte ratio, ESR: erythrocyte sedimentation rate, CRP: C-reactive protein.

**Table 3:** Comparison of FMF with amyloidosis, amyloidosis-free FMF, and controls in terms of leucocyte, erythrocyte sedimentation rate (ESR), C-reactive protein (CRP), and neutrophil/lymphocyte ratio (NLR) values.

	FMF with amyloidosis *n* = 12 (mean ± SD)	FMF without amyloidosis *n* = 82 (mean ± SD)	Controls *n* = 60 (mean ± SD)	*P* ^1^	*P* ^2^	*P* ^3^
Leucocyte, ×10^9^/L	8.35 ± 2.58	7.19 ± 1.75	6.81 ± 1.43	0.084	0.068	0.158
ESR, mm/h	24.00 ± 15.89	9.83 ± 8.42	6.40 ± 4.13	<0.0001	0.016	0.006
CRP, mg/L	4.42 ± 2.24	3.04 ± 2.08	2.43 ± 1.57	0.191	0.059	0.090
NLR, %	2.51 ± 0.62	1.99 ± 0.58	1.59 ± 0.42	0.009	<0.0001	<0.0001

*P* ^1^: *P* value comparison between FMF with amyloidosis and FMF without amyloidosis.

*P* ^2^: *P* value comparison between FMF with amyloidosis and healthy controls.

*P* ^3^: *P* value comparison between FMF without amyloidosis and healthy controls.
